# ETV2 promotes osteogenic differentiation of human dental pulp stem cells through the ERK/MAPK and PI3K-Akt signaling pathways

**DOI:** 10.1186/s13287-022-03052-2

**Published:** 2022-10-04

**Authors:** Jing Li, Haoran Du, Xin Ji, Yihan Chen, Yishuai Li, Boon Chin Heng, Jianguang Xu

**Affiliations:** 1 School of Stomatology, Shandong First Medical University and Shandong Academy of Medical Sciences, Jinan, 250000 People’s Republic of China; 2 Key Lab of Oral Diseases Research of Anhui Province, College and Hospital of Stomatology, Anhui Medical University, 69 Meishan Road, Hefei, 230032 People’s Republic of China; 3 Central Laboratory, Peking University School and Hospital of Stomatology, Beijing, 100081 People’s Republic of China

**Keywords:** ETV2, Osteogenic differentiation, Human dental pulp stem cells, Bone formation, RNA-sequencing

## Abstract

**Background:**

The repair of cranio-maxillofacial bone defects remains a formidable clinical challenge. The Ets variant 2 (ETV2) transcription factor, which belongs to the E26 transformation-specific (ETS) family, has been reported to play a key role in neovascularization. However, the role of ETV2 in the osteogenesis of human dental pulp stem cells (hDPSCs) remains unexplored.

**Methods:**

Transgenic overexpression of ETV2 was achieved using a lentiviral vector, based on a Dox-inducible system. The effects of Dox-induced overexpression of ETV2 on the osteogenesis of hDPSCs were evaluated by quantitative real-time polymerase chain reaction (qRT-PCR), western blot, immunofluorescence staining, alkaline phosphatase (ALP) staining, and Alizarin Red S (ARS) staining. Additionally, RNA-sequencing (RNA-Seq) analysis was performed to analyze the underlying mechanisms of ETV2-induced osteogenesis. Additionally, the role of ETV2 overexpression in bone formation in vivo was validated by animal studies with a rat calvarial defect model and a nude mice model.

**Results:**

Our results demonstrated that ETV2 overexpression significantly upregulated the mRNA and protein expression levels of osteogenic markers, markedly enhanced ALP activity, and promoted matrix mineralization of hDPSCs. Moreover, the results of RNA-Seq analysis and western blot showed that the ERK/MAPK and PI3K-Akt signaling pathways were activated upon transgenic overexpression of ETV2. The enhanced osteogenic differentiation of hDPSCs due to ETV2 overexpression was partially reversed by treatment with inhibitors of ERK/MAPK or PI3K-AKT signaling. Furthermore, the results of in vivo studies demonstrated that ETV2 overexpression improved bone healing in a rat calvarial defect model and increased ectopic bone formation in nude mice.

**Conclusions:**

Collectively, our results indicated that ETV2 overexpression exerted positive effects on the osteogenesis of hDPSCs, at least partially via the ERK/MAPK and PI3K/AKT signaling pathways.

**Supplementary Information:**

The online version contains supplementary material available at 10.1186/s13287-022-03052-2.

## Introduction

Reconstruction of cranio-maxillofacial bone defects remains a formidable clinical problem to date [1]. Cranio-maxillofacial bone defects can be caused by tumors, trauma, inflammation, and congenital malformations, leading to reduced quality of life, such as disorders of chewing or swallowing. Considered as the gold standard, autogenous bone grafts are known to be safe and clinically effective, but their clinical applications are limited due to various complications including infection, lack of donor sites, and secondary damage [2, 3]. In recent years, bone tissue engineering has emerged as a promising alternative for treatment of bone defects [4].

Mesenchymal stem cells (MSCs) are widely regarded to be promising cell sources for bone tissue engineering, due to their multi-lineage differentiation potential and extensive self-renewal capacity [5]. As a type of MSCs derived from dental tissues, human dental pulp stem cells (hDPSCs) are easily and readily isolated from extracted teeth. Notably, hDPSCs have been reported to exhibit a similar or even higher osteogenic potential compared with bone marrow mesenchymal stem cells (BMSCs) [6–9]. Numerous previous studies have reported that the osteogenic capacity of MSCs can be regulated by many factors [10, 11], including noncoding RNA, transcription factors, bioactive materials, and various different biophysical stimuli.

Previous studies have reported that the E26 transformation-specific (ETS) family of transcription factors are implicated in a diverse array of cellular activities [12, 13], such as cell proliferation, migration, apoptosis, and cell differentiation. Most notably, several members of the ETS family, including ELF4 [14], ETS1, and ETS2 [15], have been reported to regulate osteogenesis. Also, increasing evidence has shown that ETS transcription factors play key roles in endothelial cell development [16–18]. In particular, the ETS factor, Ets variant 2 (ETV2), is closely linked to development of the vascular lineage [19]. Likewise, in our previous study, we found that vasculogenesis of hDPSCs was upregulated through recombinant ETV2 overexpression [20]. However, no study has yet reported the effects of ETV2 overexpression on the regulation of bone metabolism.

The objective of this study was to therefore explore the effects of ETV2 overexpression on the osteogenesis of hDPSCs. The upregulated expression of osteogenic markers and enhanced alkaline phosphatase (ALP) activity and matrix mineralization in this study demonstrated that ETV2 was a positive regulator of hDPSCs during osteogenic differentiation. Mechanistically, we demonstrated that ETV2 overexpression increased osteogenic differentiation of hDPSCs via the ERK/MAPK and PI3K-Akt signaling pathways. Additionally, our in vivo studies showed that ETV2 overexpression improved bone healing in a rat calvarial defect model and enhanced ectopic bone formation in nude mice.

## Methods

### Cell culture

Human dental pulp tissues were obtained from three healthy donors at the Stomatological Hospital of Shandong University, with written consent from all donors. hDPSCs were isolated as previously described [20]. Cells were then cultured in a-MEM supplemented with 10% (v/v) fetal bovine serum. For validation of “stemness,” flow cytometry was used to analyze cell surface marker expression by hDPSCs and the cells were also incubated in osteogenic, adipogenic, and chondrogenic induction medium as previously described [20]. All in vitro assays were performed at least 3 times.

### Lentivirus transduction

ETV2-encoding lentivirus and control lentivirus based on the Dox-inducible system were purchased from GeneChem Company (Shanghai, China). Both lentiviral particles were used to transduce passage 3 hDPSCs with 1× HitransG A transfection enhancer. At 12 h after transduction, the transfection medium was removed. Following selection with antibiotics, Dox was used to induce the expression of ETV2 in hDPSCs transfected by ETV2-encoding lentivirus at various concentrations (0, 50, 100, and 200 ng/ml). Thereafter, the optimum concentration of Dox was determined to be 100 ng/ml using quantitative real-time polymerase chain reaction (qRT-PCR) and western blot analysis. Further validation of 100 ng/ml Dox induction on ETV2 expression was performed through qRT-PCR, western blot, and immunofluorescence assays.

Based on transfection by ETV2-encoding lentivirus or control lentivirus and the presence or absence of Dox, hDPSCs were divided into 4 groups, including NC-Dox (−), NC-Dox (+), ETV2-Dox (−), and ETV2-Dox (+).

### Cell counting kit-8 (CCK-8) assay

To assess the effects of ETV2 overexpression on the proliferation of hDPSCs, transfected cells were seeded into 96-well plates at a plating density of 1000 cells per well. 1, 3, 5, and 7 days later, hDPSCs were incubated with 10 μL CCK-8 reagent (Dojindo, Kumamoto, Japan), at 37 °C for 2 h. Finally, absorbance was measured at 450 nm.

### qRT-PCR

Total RNA was extracted from transfected hDPSCs using Trizol reagent (Invitrogen, NY, USA). Following determination of RNA concentration with Nanodrop, cDNA was synthesized using a PrimeScript RT reagent Kit (TaKaRa Biotech, Tokyo, Japan) in accordance with manufacturers' instructions. Following qRT-PCR using the Roche 480 instrument, the 2^−△△Ct^ method was used to determine the relative expression levels of various gene transcripts. Glyceraldehyde-3-phosphate dehydrogenase (GAPDH) was selected to be the reference housekeeping gene. All primer sequences are listed in Table 1.

**Table 1:** Sequences of the primers

Gene	Primers	Sequence
ETV2	Forward	ACGTCTCGGAAAATTCCCCC
	Reverse	CATCCCAGTTCCACAGGTCC
ALP	Forward	TTGACCTCCTCGGAAGACACTCTG
	Reverse	CGCCTGGTAGTTGTTGTGAGCATAG
COL1A1	Forward	GAGAGCATGACCGATGGATT
	Reverse	CCTTCTTGAGGTTGCCAGTC
OSX	Forward	CCTGCGACTGCCCTAATT
	Reverse	GCGAAGCCTTGCCATACA
GATA2	Forward	GCTCGTTCCTGTTCAGAAGGC
	Reverse	CCCATTCATCTTGTGGTAGAGGC
IGFBP5	Forward	ACCTGAGATGAGACAGGAGTC
	Reverse	GTAGAATCCTTTGCGGTCACAA
TLR2	Forward	TTATCCAGCACACGAATACACAG
	Reverse	AGGCATCTGGTAGAGTCATCAA
APOD	Forward	ATCCAGGCCAACTACTCACT
	Reverse	GATTCACAGTTCCATCAGCT
EphB6	Forward	TGCTGGTGAATAGCCACTTG
	Reverse	CGGAACTCCTGCTCTATTGC
FBXO32	Forward	GAAGCGCTTCCTGGATGAGA
	Reverse	GGAATCCAGAATGGCAGTTG
GAPDH	Forward	TGCACCACCAACTGCTTAGC
	Reverse	GGCATGGACTGTGGTCATGAG

### Western blot analysis

Total protein lysates were harvested with RIPA lysis buffer (Beyotime, Shanghai, China) supplemented with PMSF (Beyotime) and protease inhibitor (Beyotime). Following electrophoresis on 10% (w/v) SDS–polyacrylamide gels, the samples were then transferred onto PVDF membranes (Millipore, Temecula, USA). Next, the blotted membranes were blocked at room temperature for 1 h, followed by incubation with primary antibodies against ETV2 (Abcam, MA, USA), ALP (Abcam), COL1A1 (Wanlei, Shenyang, China), OSX (Abcam), t-ERK (CST, MA, USA), p-ERK (CST), t-AKT (CST), p-AKT (CST), and GAPDH (Abcam) overnight at 4 °C. Subsequently, samples were incubated with secondary antibodies (Zsbio, Beijing, China) for 1 h. The bands were visualized using an ECL Kit (Millipore).

### Immunocytochemistry

Transfected hDPSCs were treated with 4% (w/v) paraformaldehyde for 0.5 h and then permeabilized using 0.25% (w/v) Triton X-100 for 10 min. After blocking for 1 h, hDPSCs were incubated with anti-ETV2 (Abcam) and OSX (Abcam) overnight. Subsequently, hDPSCs were incubated with the fluorescently labeled secondary antibody for 1 h. Finally, the DNA was stained with diamidinophenylindole, and then, the samples were observed under fluorescence microscopy (Olympus, Tokyo, Japan).

### ALP staining and activity

At 7 and 14 days after osteogenic induction, the transfected hDPSCs were rinsed with phosphate-buffered saline (PBS) and then fixed in 4% (w/v) paraformaldehyde. Subsequently, hDPSCs were treated with BCIP/NBT staining kit (Beyotime). Measurement of ALP activity was conducted using the ALP Activity Assay Kit (Beyotime) with absorbance being detected at 520 nm.

### Alizarin Red S (ARS) staining

At 14 and 21 days of osteogenic differentiation, hDPSCs were rinsed in PBS for 3 times. Subsequently, after being fixed, hDPSCs were incubated with 1% ARS solution for 15 min. To assess the degree of mineral deposition, the stain was desorbed using cetylpyridinium chloride (Sigma-Aldrich, St. Louis, USA), with the absorbance being quantified at 562 nm.

### RNA-sequencing (RNA-Seq) analysis

Following ETV2-encoding lentivirus transfection, hDPSCs were incubated in osteogenic differentiation medium with or without Dox for 10 days. Subsequently, total RNAs were extracted, used to generate cDNA libraries, and then sequenced using an Illumina Novaseq™ 6000 (LC-Bio Technology CO., Ltd., Hangzhou, China). Using the DESeq2 method, genes that were upregulated or downregulated more than twofold between the Dox (+) and Dox (−) groups were referred to as differentially expressed genes. The identified genes were used for gene ontology (GO) and Kyoto Encyclopedia of Genes and Genomes (KEGG) pathways analysis. All data have been deposited into the Gene Expression Omnibus database and are available with the accession number GSE160451.

### Animal experiments

β-TCP scaffolds (Φ5 × 2 mm^3^, Sichuan University, Chengdu, China) with an average porosity of 75% were used in our animal experiments. After osteogenic differentiation for 7 days, hDPSCs were trypsinized, resuspended, and then attached to β-TCP scaffolds (approximately 2 × 10^6^ cells per scaffold). Next, the hDPSCs/β-TCP complexes were incubated at 37 °C for 4 h before implanted in vivo. Based on various treatments of hDPSCs, the complexes were divided into 4 groups: NC-Dox (−), NC-Dox (+), ETV2-Dox (−), and ETV2-Dox (+). To construct the calvarial defect model, 24 male 8-week-old SD rats (*n* = 6 per group) were anesthetized using 3% (w/v) pentobarbital sodium. After making a linear incision in the sagittal direction to uncover the skull, a 5-mm-diameter craniotomy defect was prepared with a trephine on both sides of the skull. Then, the hDPSCs/β-TCP complexes were placed in the defect area. To evaluate the effects of ETV2 overexpression on ectopic osteogenesis of hDPSCs, the complexes were also implanted subcutaneously at the back of 16 male 8-week-old athymic nude mice (*n* = 4 per group).

### Analysis of bone regeneration in vivo

8 weeks after operation, all animals were euthanized and the specimens obtained were then fixed in 4% (w/v) paraformaldehyde. Specifically, the rat skulls were scanned using a micro-CT system (GE Healthcare Biosciences, Pittsburgh, USA) in high-resolution mode (voltage, 80 kV; current, 80 μA; pixel size, 15 μm). After scanning, the GEHC MicroView software (GE Healthcare Biosciences) was used to reconstruct 3D images of the rat skulls. The bone mineral density (BMD), bone volume/total volume (BV/TV), and trabecular thickness (Tb.Th) were analyzed for each group. Then, all specimens were decalcified, followed by wax immersion. Thereafter, hematoxylin and eosin (HE) staining and Masson staining of all serial sections were carried out.

### Statistical analysis

All data were presented as mean + SD. The Prism 6.0 software (GraphPad Software, La Jolla, USA) was used for statistical comparisons of our results. Student’s t test and ANOVA were applied to paired and multiple comparisons, respectively. Values of *P* < 0.05 were considered significant.

## Results

### Induction of transgenic ETV2 overexpression by Dox in hDPSCs

**Fig. 1:**
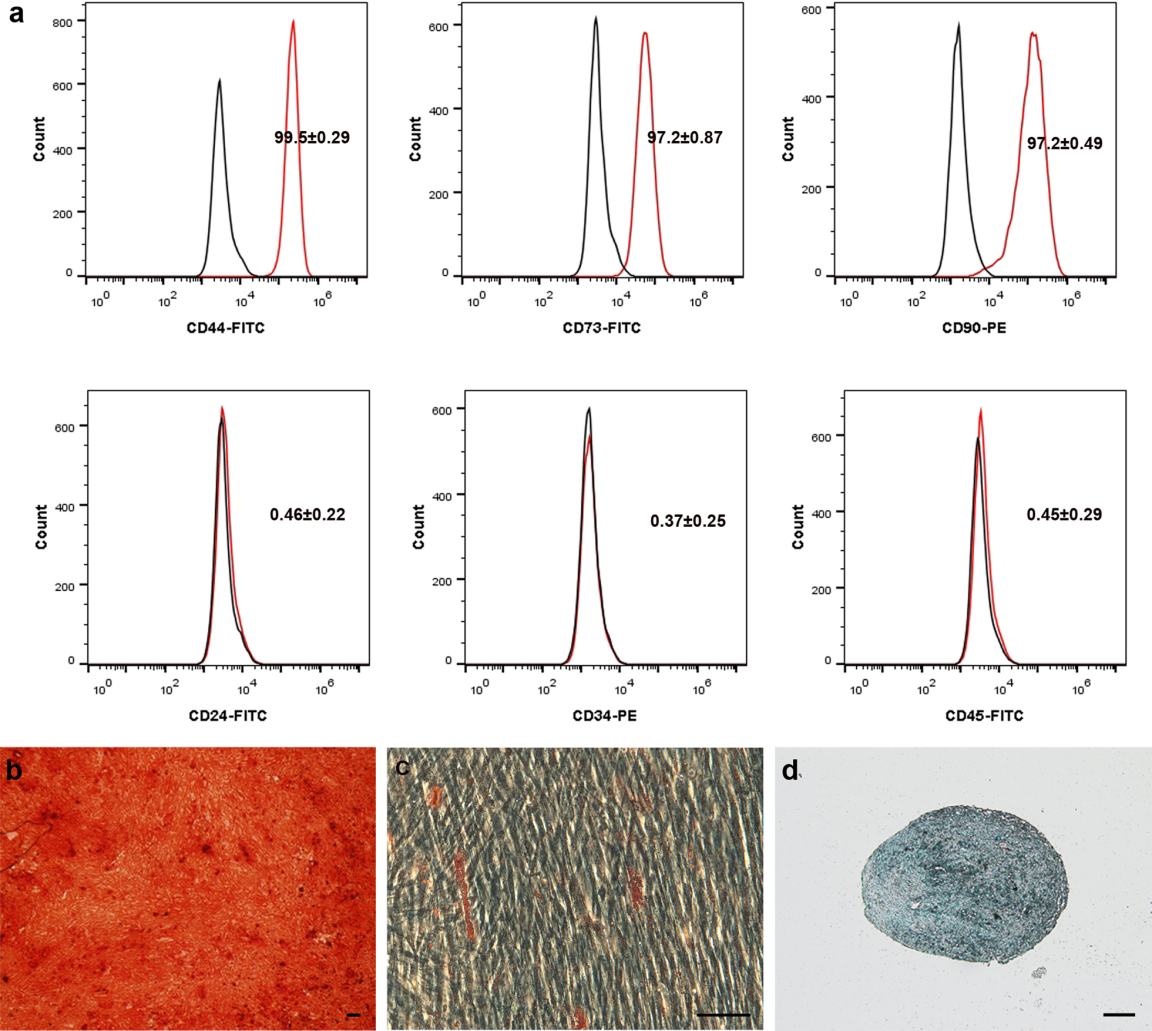
Characterization of hDPSCs. **a** Verification of cell surface antigen expression by flow cytometry. The differentiation potential of hDPSCs into the osteo-, adipo-, and chondrogenic lineages was verified through ARS (**b**), Safranin O (**c**), and Oil red O (**d**) stains, respectively. Scale bar: 100 μm

Flow cytometry analysis of hDPSCs revealed that hDPSCs were positive for expression of the canonical MSC markers CD44, CD73, and CD90, while being negative for expression of the hematopoietic markers CD24, CD34, and CD45. Meanwhile, as shown in Fig. 1, the results of ARS, Safranin O, and Oil red O stains validated the multi-lineage differentiation potential of hDPSCs into osteoblasts, adipocytes, and chondroblasts, respectively.

**Fig. 2:**
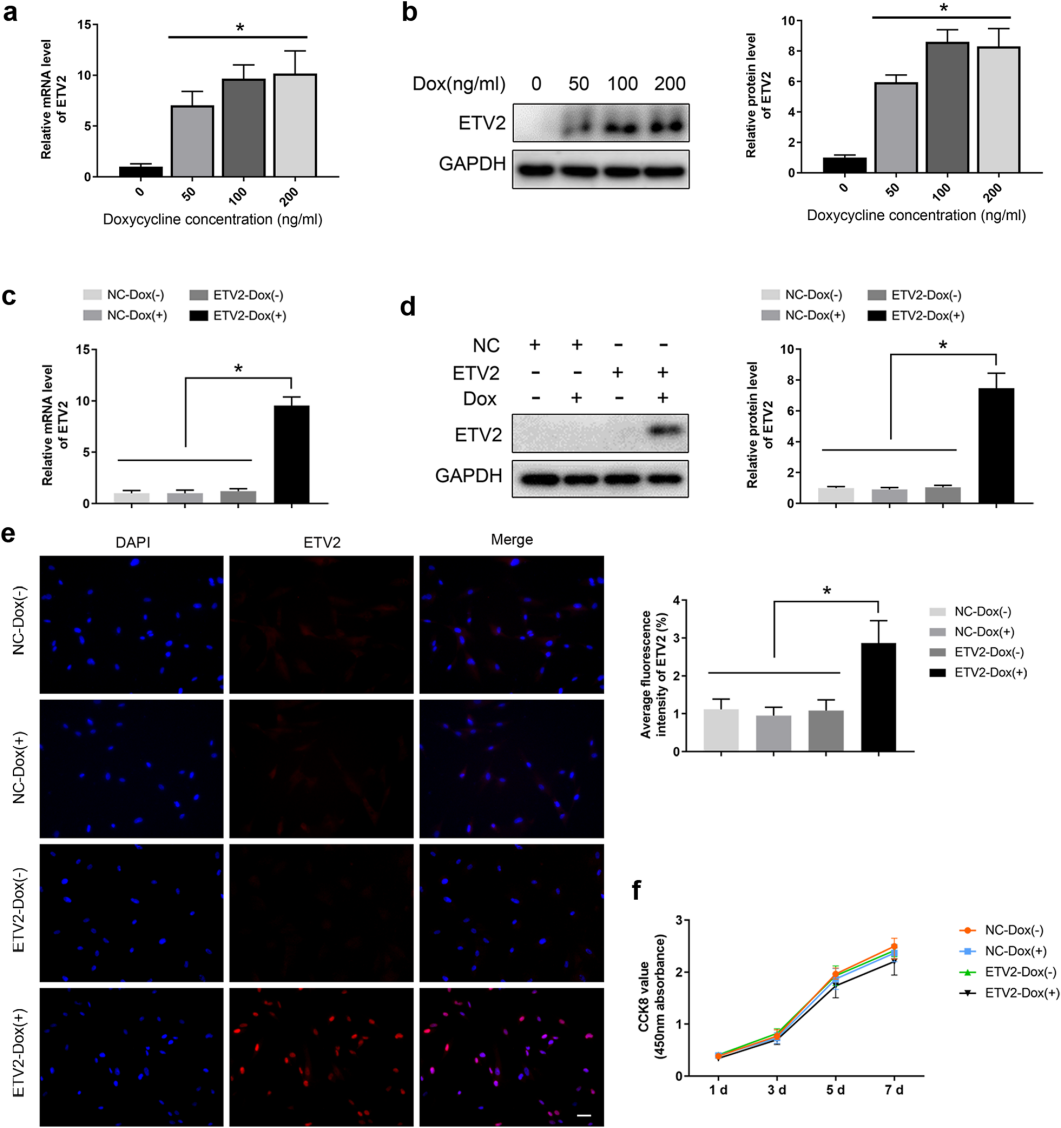
Verification of Dox-inducible overexpression of ETV2 in hDPSCs. **a, b** The optimal concentration of Dox was determined by qRT–PCR (**a**) and western blot (**b**). **c-e** Induction of ETV2 expression by Dox (100 ng/mL) at the mRNA and protein levels was measured by qRT–PCR (**c**), western blot (**d**), and immunofluorescence staining (**e**). **f** Detection of hDPSCs proliferation at days 1, 3, 5, and 7 by CCK-8 assay. Error bars, mean ± SD (*n* = 3). * *P* < 0.05. Scale bar: 50 μm

The Dox-inducible system was used in this study to induce transgenic ETV2 overexpression in hDPSCs. Initially, the optimum concentration of Dox was identified by qRT-PCR and western blot analysis. After transfection by ETV2-encoding lentivirus, the mRNA and protein expression levels of ETV2 in hDPSCs treated with Dox were more than fivefold higher compared to hDPSCs without Dox treatment. Transfected hDPSCs with 100 and 200 ng/ml Dox treatment showed higher ETV2 expression than the 50 ng/ml Dox group, while the difference between the 100 and 200 ng/ml Dox groups was not statistically significant (Fig. 2a, b). Hence, we chose the dose of 100 ng/ml Dox for further experiments.

Thereafter, hDPSCs were transduced with ETV2-encoding lentivirus or control lentivirus with or without Dox. As shown in Fig. 2c, the mRNA expression of ETV2 in the ETV2-Dox (+) group was significantly higher than that in the NC-Dox (−), NC-Dox (+), and ETV2-Dox (−) groups. Similarly, upregulated expression of ETV2 at the protein level was also detected in the ETV2-Dox ( +) group, and differences between the ETV2-Dox (+) group and the other 3 groups were statistically significant (Fig. 2d, e).

### ETV2 overexpression had no effect on hDPSCs proliferation

The effects of ETV2 overexpression on hDPSCs proliferation on days 1, 3, 5, and 7 are shown in Fig. 2f. The viability of hDPSCs in the ETV2-Dox (+) group was slightly reduced on days 5 and 7, but the differences between the groups at all time points were not statistically significant, indicating that ETV2 overexpression might not have any impact on hDPSCs proliferation.

### ETV2 overexpression enhanced osteogenic differentiation of hDPSCs

**Fig. 3:**
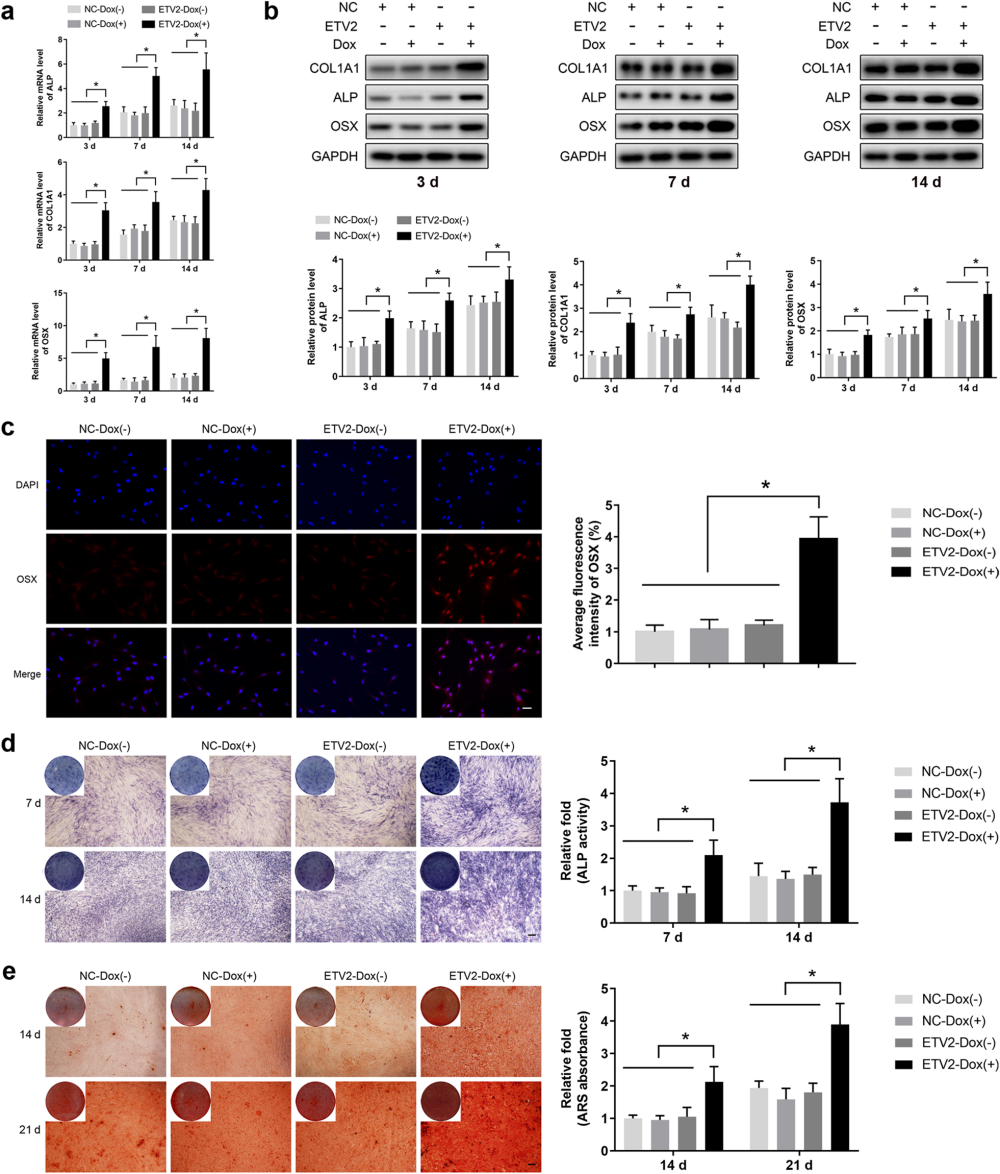
Transgenic ETV2 overexpression enhanced osteogenic differentiation of hDPSCs. **a, b** The expression of osteo-specific markers including ALP, COL1A1, and OSX was analyzed using qRT–PCR (**a**) and western blot (**b**). **c** Detection of osteogenic marker OSX at day 3 after osteogenic induction by immunocytochemistry and relative quantitative analysis. Scale bar: 50 μm. **d** ALP staining was conducted at days 7 and 14 of osteogenic differentiation. Scale bar: 200 μm. Histograms showing quantitative analysis of ALP activity. **e** The effects of ETV2 overexpression on calcium deposition were assessed by ARS staining at days 14 and 21 of osteogenic induction. Scale bar: 200 μm. Histograms showing quantitative data. Error bars, mean ± SD (*n* = 3). * *P* < 0.05

To further clarify the role of ETV2 overexpression in osteogenic differentiation of hDPSCs, mRNA expression of the osteogenic markers ALP, COL1A1, and OSX was detected using qRT–PCR. After osteogenic induction for 3, 7, and 14 days, the results of qRT–PCR showed that significantly increased expression of osteoblast differentiation-related genes was only detected in the ETV2-Dox (+) group (Fig. 3a). Similarly, western blot analysis revealed that protein expression levels of ALP, COL1A1, and OSX in the ETV2-Dox (+) group were significantly higher in the ETV2-Dox (+) group than in the NC-Dox (−), NC-Dox (+), or ETV2-Dox (−) groups (Fig. 3b).

As an osteoblast-specific transcription factor, OSX plays a pivotal role in regulating osteogenic differentiation and determines the expression levels of multiple osteoblastic markers. Therefore, OSX was selected for investigation by immunocytochemistry. Of note, upregulated expression of OSX protein was detected in ETV2-overexpressing cells on day 3 after osteogenic induction with immunocytochemistry (Fig. 3c).

On days 7 and 14 of osteogenesis, the ETV2-Dox (+) group displayed enhanced ALP activity compared to the NC-Dox (−), NC-Dox (+), and ETV2-Dox (−) groups (Fig. 3d). Moreover, ARS staining was used to assess calcium deposit formation after osteogenic induction for 14 and 21 days. More matrix mineralization was observed in ETV2-overexpressing hDPSCs, as compared to cells in the other 3 groups (Fig. 3e). Collectively, our results proved that recombinant ETV2 overexpression exerts positive effects on the osteogenic differentiation of hDPSCs.

### ETV2 overexpression activated the ERK/MAPK and PI3K-Akt signaling pathways

**Fig. 4:**
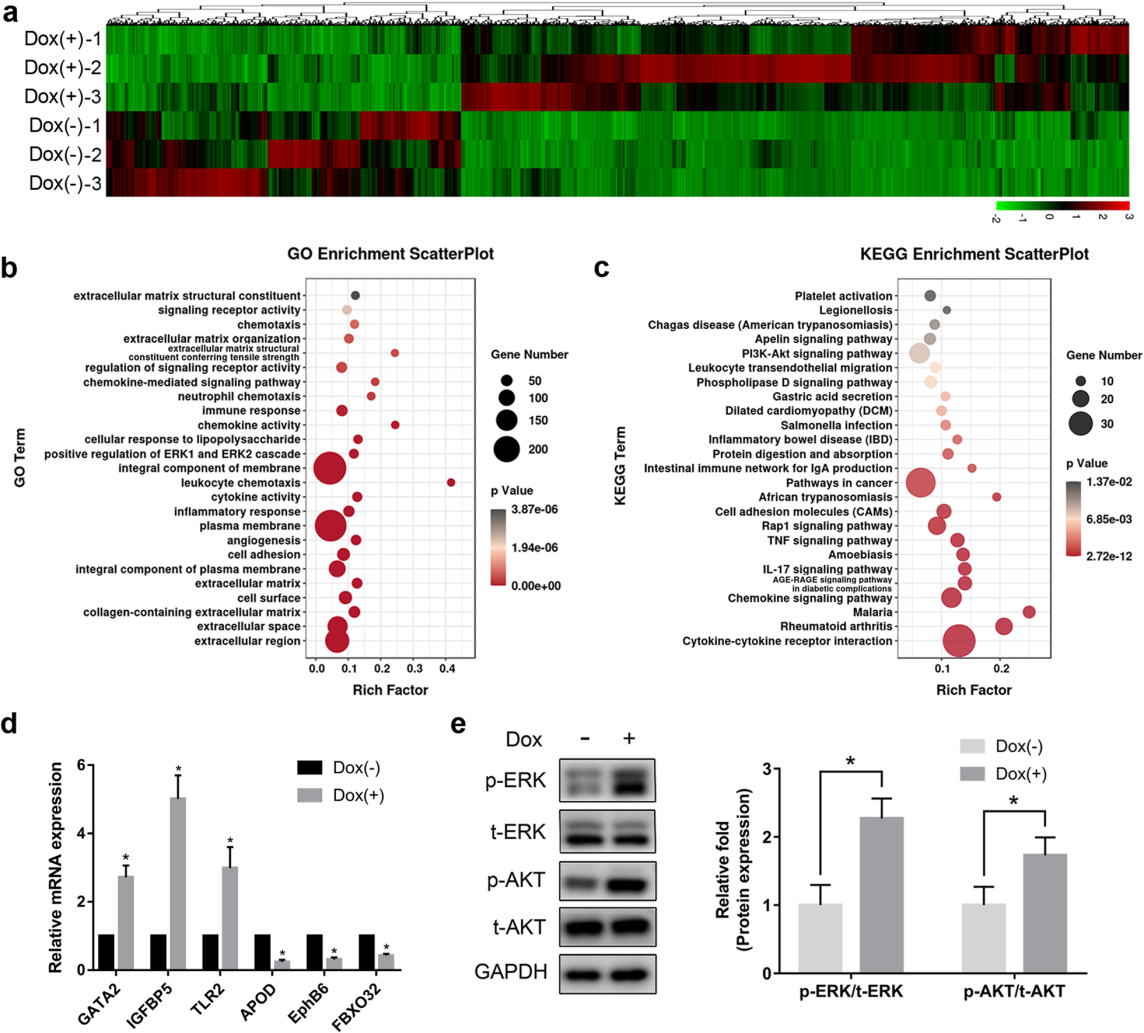
RNA‐Seq analysis of hDPSCs after osteogenic induction for 10 days. **a** Heatmap of 1121 differentially expressed genes in hDPSCs transfected by ETV2-encoding lentivirus, with or without Dox treatment. **b, c** Bubble plots showing GO (**b**) and KEGG pathway (**c**) enrichment analysis of the differentially expressed genes. **d** qRT–PCR validation of 3 upregulated genes and 3 downregulated genes in RNA‐Seq results. **e** Results of western blot analysis showed that ETV2 overexpression increased the level of phosphorylated ERK and AKT in hDPSCs. Error bars, mean ± SD (*n* = 3).* *P* < 0.05

To further explore the underlying mechanisms of the regulatory effects of ETV2 overexpression on osteogenesis, RNA-Seq was performed. Among the identified genes, 732 upregulated and 389 downregulated genes were found in transfected hDPSCs with Dox treatment, as compared to the control cells without Dox treatment, using the DESeq2 method (Fig. 4a). Next, GO enrichment analysis was performed on the 1121 differentially expressed genes. As presented in Fig. 4b, the top 25 enriched GO terms include “collagen-containing extracellular matrix,” “angiogenesis,” “positive regulation of ERK1 and ERK2 cascade,” and “regulation of signaling receptor activity.” In particular, the term “positive regulation of ERK1 and ERK2 cascade” indicated that the ERK/MAPK pathway may be associated with regulation of osteogenic differentiation by ETV2 overexpression. Meanwhile, Fig. 4c shows the top 25 KEGG pathways and analysis on KEGG pathway enrichment demonstrated that the “PI3K-Akt signaling pathway” may be involved in ETV2-mediated osteogenesis. Subsequently, qRT–PCR was performed to validate the RNA-Seq results of 3 upregulated genes (GATA2, IGFBP5, and TLR2) and 3 downregulated genes (APOD, EphB6, and FBXO32). As shown in Fig. 4d, the results of qRT–PCR revealed that those genes displayed similar expression trends as the RNA-Seq data. Interestingly, the mRNA level of CBFA1 in qRT-PCR (Additional file 1: Fig. S1a) and RNA-Seq (Additional file 1: Fig. S1b) was almost unchanged or slightly reduced in the Dox (+) group when compared to the Dox (−) group. Furthermore, the potential pathways activated by ETV2 expression were verified by western blot. On day 3 after osteogenic induction, upregulated phosphorylation levels of ERK and Akt were found in hDPSCs with Dox treatment, while no significant changes were detected in the expression levels of t-ERK and t-Akt (Fig. 4e), indicating that ETV2 overexpression led to activation of the ERK/MAPK and PI3K-Akt signaling pathways during osteogenic differentiation. Notably, the activation of p-ERK and p-AKT still can be detected at days 7 and 14 of osteogenic differentiation (Additional file 2: Fig. S2).

### Inhibiting the ERK/MAPK and PI3K-Akt signaling pathways partially reversed the stimulatory effects of ETV2 overexpression on the osteogenesis of hDPSCs

**Fig. 5:**
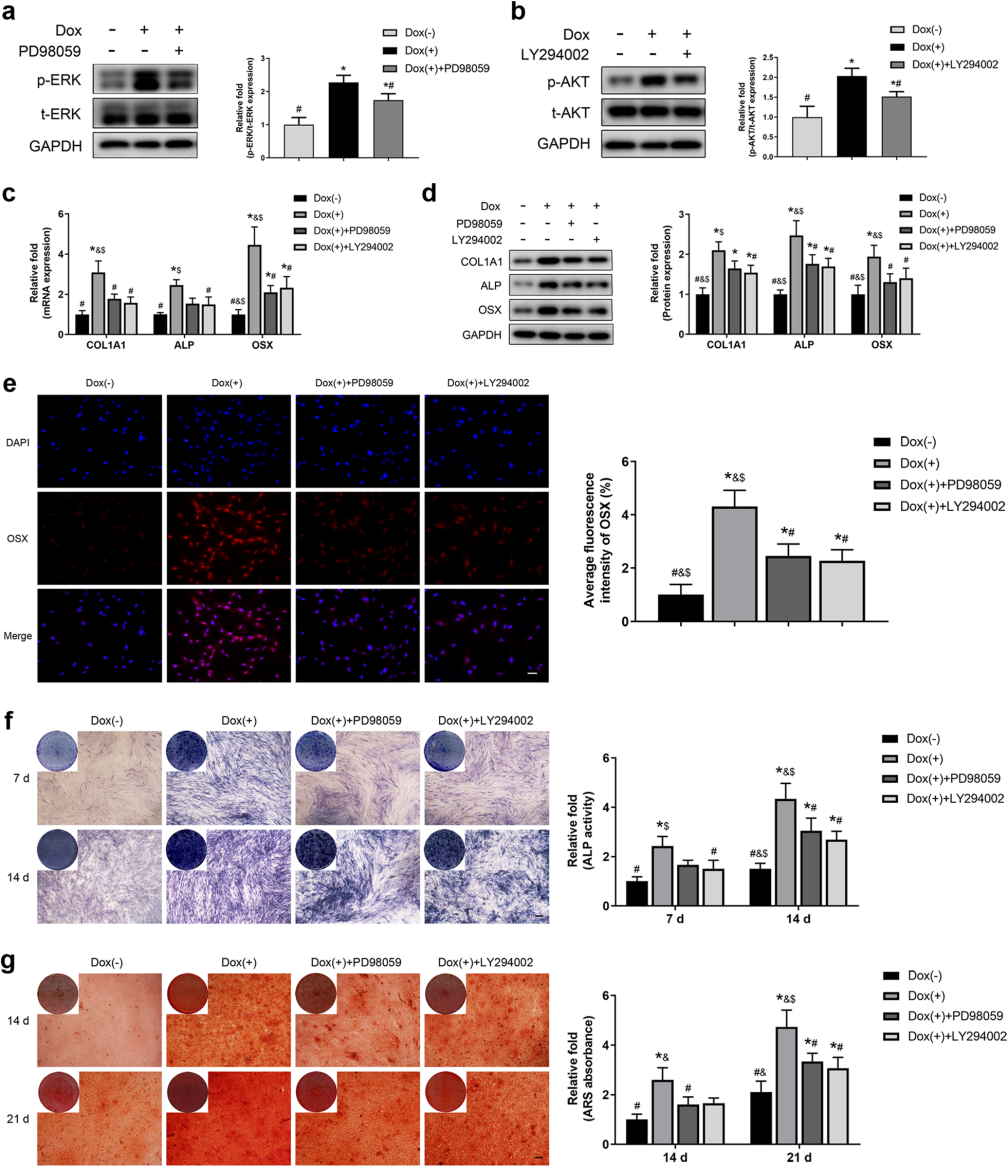
ERK and AKT inhibition decreased the enhancing effect of ETV2 overexpression on osteogenesis of hDPSCs. **a, b** The addition of 50 μM PD98059 (**a**) or 20 μM LY294002 (**b**) reduced the levels of phosphorylated ERK (**a**) or AKT (**b**) due to ETV2 overexpression. **c, d** Results of qRT–PCR (**c**) and western blot analysis (**d**) revealed that PD98059 and LY294002 partially decreased the upregulated mRNA and protein expression of osteogenic markers due to ETV2 overexpression. **e** Representative images of immunofluorescence staining of OSX at day 3 after osteogenic induction and corresponding quantification. Scale bar: 50 μm. **f** ALP staining after osteogenic induction for 7 and 14 days and relative quantification of ALP activity. Scale bar: 200 μm. **g** ARS staining at days 14 and 21 of osteogenic induction and corresponding quantitative analysis. Scale bar: 200 μm. * P < 0.05, compared with DOX (−-) group; #* P* < 0.05, compared with DOX (+) group; &* P* < 0.05, compared with DOX (+) + PD98059 group; $* P* < 0.05, compared with DOX (+) + LY294002 group. Error bars, mean ± SD (*n* = 3)

To determine whether the ERK/MAPK and PI3K-Akt signaling pathways played a role in the enhancing effect of ETV2 overexpression on osteogenic differentiation, inhibitors of the ERK/MAPK signaling pathway (50 μM PD98059) [21, 22] and PI3K-Akt signaling pathway (20 μM LY294002) [23, 24] were used in this study. After 3 days of osteogenic induction, as shown in Fig. 5a and b, the addition of PD98059 and LY294002 suppressed phosphorylation of the ERK/MAPK signaling pathway and PI3K-Akt signaling pathway, respectively. Consistent with decreased expression levels of p-ERK and p-AKT, the upregulated mRNA and protein expression of ALP, COL1A1, and OSX in ETV2-overexpressing hDPSCs was partially reversed in the presence of signaling inhibitors (Fig. 5c, d). Likewise, the immunocytochemistry results showed that the addition of PD98059 and LY294002 partially lessened the enhanced OSX protein expression level induced by ETV2 overexpression (Fig. 5e).

Moreover, the upregulated ALP activity induced by ETV2 overexpression was partially reduced after 7 and 14 days of osteogenic differentiation (Fig. 5f). The same trend was observed in ARS staining at days 14 and 21 of osteogenic differentiation (Fig. 5g).

### ETV2 overexpression promoted bone formation in vivo

**Fig. 6:**
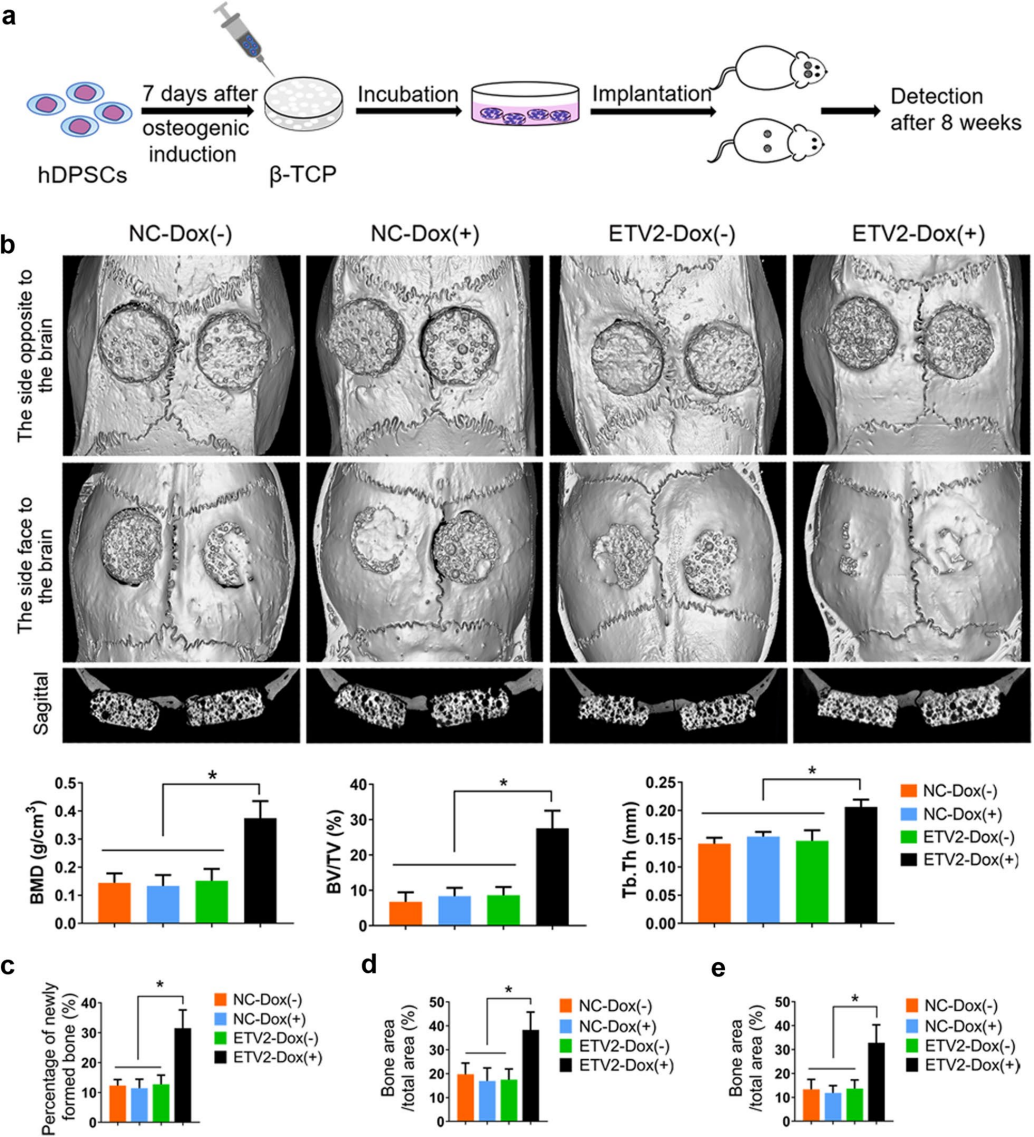
ETV2 overexpression enhanced the osteogenic potential of hDPSCs in vivo. **a** Schematics of the experimental process. **b** Micro-CT scanning of rat calvarial defects (*n* = 6). Upper panel: Representative images of Micro-CT scanning at 8 weeks post-surgery; Lower panel: Quantitative analysis of BMD, BV/TV, and Tb.Th. **c-e** Quantitative analysis of newly formed bone upon HE staining of the rat calvarial defect model (**c**), and HE (**d**) and Masson (**e**) staining in mice model. Error bars, mean ± SD. * *P* < 0.05

In light of the favorable effects of ETV2 overexpression on the osteogenesis of hDPSCs, we hypothesized that Dox-inducible overexpression of ETV2 may also possess the potential to improve bone formation in vivo. The schematic diagram illustrated how the hDPSCs/β-TCP complex was prepared and employed in the rat calvarial defect model and nude mice model (Fig. 6a). After 8 weeks, all samples were harvested and analyzed. 3D reconstruction of the rat calvaria using micro-CT revealed that the appearance of the implants was smoother with more newly formed bone being observed in the ETV2-Dox (+) group versus the other 3 groups. In addition, quantitative analysis showed that BMD, BV/TV, and Tb.Th parameters in the ETV2-Dox (+) group were significantly higher, as compared to the NC-Dox (−), NC-Dox (+), and ETV2-Dox (−) groups (Fig. 6b).

Furthermore, histological examination of the undecalcified samples corroborated the radiographic data. Microscopically, the largest area of newly formed bone was found in the ETV2-Dox (+) group (Additional file 3: Fig. S3a) and the maturity of the newly formed bone in the ETV2-Dox (+) group was significantly better than in the other 3 groups (Additional file 3: Fig. S3b). Meanwhile, the results of HE and Masson staining showed that the formation of organized bone matrix and fibrous tissue was detected in the mice model. Similarly, the ETV2-Dox (+) group displayed the most amount of bone matrix (Additional file 4: Fig. S4a, b). Additionally, quantitation of Masson staining in the rat calvarial defect model showed that the percentage of newly formed bone was 12.27 ± 1.95% in the NC-Dox (−) group, 11.44 ± 2.86% in the NC-Dox (+) group, 12.76 ± 2.91% in the ETV2-Dox (−) group, and 31.54 ± 5.85% in the ETV2-Dox (+) group, respectively (Fig. 6c). The differences between the ETV2-Dox (+) group versus the other 3 groups were statistically significant. Consistently, quantitative analysis of HE and Masson staining in the mice model showed the same tendency (Fig. 6d, e). Altogether, our results indicated that ETV2 overexpression plays an active role in bone formation in vivo.

## Discussion

Various methods of bone reconstruction have been adopted to repair cranio-maxillofacial bone defects [1, 25, 26]. In recent years, a growing number of studies indicated that bone tissue engineering is a viable alternative to traditional autogenous, allogenic, and xenogenic bone grafts [27, 28]. In this study, hDPSCs were utilized as seed cells. As MSCs from dental pulp tissue, hDPSCs have the potential to differentiate into osteoblasts and can be regarded as potential stem cell candidates for bone tissue engineering. In parallel, previous studies showed that genetic manipulation was often used to stimulate the osteogenic potential of stem cells [29, 30]. In this study, we induced transgenic overexpression of ETS transcription factor ETV2 in hDPSCs, as a novel strategy for bone regeneration.

Lentiviral vectors have been widely used as efficient gene delivery vehicles to transfect various different types of MSCs. In this study, they were chosen to induce stable overexpression of ETV2 in DPSCs. However, it has been reported that ETV2 may lead to excessive angiogenesis, which is highly correlated with malignancy [31]. To avoid the potential risk of tumorigenesis, we adopted the Dox-inducible Tet-On system to regulate recombinant ETV2 overexpression. In this study, we induced target gene expression by addition of 100 ng/mL Dox, similar to the previous study of Tóth et al. [32]. Subsequently, controllable ETV2 overexpression was achieved in hDPSCs upon transfection by Tet-On lentiviruses carrying human ETV2 in the presence of Dox. Notably, the background expression of ETV2 was extremely low in transfected hDPSCs without Dox treatment. Moreover, the results of the CCK-8 assay showed no significant effects on the proliferation of hDPSCs treated with control lentivirus in the presence or absence of Dox, implying that Dox had no detrimental side effects on the mitosis of hDPSCs.

A variety of different approaches have been reported to enhance the osteogenic lineage differentiation of MSCs. Safari et al. [33] reported that different bioactive agents have been used in bone tissue engineering to accelerate bone regeneration, such as organic small molecules and drugs, bioceramics, or metallic nanoparticles. Additionally, numerous studies have suggested that noncoding RNAs could function as key regulators of osteogenic differentiation. According to a study by Li et al. [34], lncRNA-H19 sponging miRNA-141 could modulate osteogenesis in nude mice models as well as in stem cells from apical papilla cultured *in vitro*. Interestingly, the expression of noncoding RNAs during osteogenic differentiation can be regulated by different biophysical stimuli, such as mechanical strain, fluid shear stress, or microgravity, followed by upregulation or downregulation of osteoblast differentiation-related genes [11]. Additionally, increasing evidence has demonstrated that osteogenic differentiation of MSCs can be genetically manipulated by various transcription factors, such as myocardin-related transcription factor A [35], zinc finger E-box-binding homeobox 1 [36], and T-Box20 [37].

ETV2 belongs to the ETS family of transcription factors. In our previous study, ETV2 has been demonstrated to enhance angiogenesis of hDPSCs [20]. Yet, the effects of ETV2 on osteogenesis have not yet been investigated. Hence, this study explored the role of ETV2 in osteogenesis. Our results demonstrated that the expression levels of osteogenesis-related genes were enhanced due to ETV2 overexpression. Specifically, OSX, the master transcription factor regulating osteogenic differentiation of MSCs [38], was significantly upregulated when ETV2 expression was activated. Moreover, ETV2 overexpression markedly enhanced ALP activity and mineral deposition of hDPSCs. Taken together, these results thus indicated that ETV2 overexpression exerts stimulatory effects on osteogenic differentiation of hDPSCs.

In this study, RNA-Seq was used to further explore the possible molecular mechanisms that contribute to the observed enhancement of osteogenic differentiation by ETV2 overexpression. Bioinformatic analyses of our RNA-Seq data revealed that the ERK/MAPK and PI3K-Akt signaling pathways could be associated with this biological process. Previous studies have reported that both pathways are closely associated with osteogenic differentiation. Zeng et al. [39] showed that chrysin can promote osteogenic differentiation via activation of the ERK/MAPK pathway. Ye et al. [23] found that the PI3K/AKT signaling pathway was activated when IL-37 upregulated osteogenesis of human BMSCs, and that the inhibitor of PI3K/AKT signaling pathway could partly reverse the enhancement of osteogenesis. Moreover, in a study by Yan et al. [40], the transcriptome analysis of human skeletal muscle cells after ETV2 overexpression revealed that the VEGF-VEGFR2 and PI3K/AKT signaling pathways were activated. Also, increased VEGF/VEGF receptor expression has been reported to be related to the PI3K-Akt and ERK/MAPK signaling pathways [41]. In parallel, increased phpspho-ERK1/2 expression was detected when ETV2 overexpression was induced in differentiating mouse embryonic stem cells, and the addition of ERK/MAPK inhibitor could partly abrogate ETV2-induced FLK1+ cell generation [42]. In our current study, we found that ETV2 overexpression activated the ERK/MAPK and PI3K-Akt signaling pathways during osteogenic differentiation of hDPSCs. Furthermore, the presence of inhibitors of the ERK/MAPK and PI3K-Akt signaling pathways both markedly attenuated ETV2–induced osteogenesis. These findings thus indicate that ETV2 overexpression promotes osteogenesis of hDPSCs, at least partly through activation of the ERK/MAPK and PI3K-Akt signaling pathways.

The effects of ETV2 overexpression on bone formation were validated via a rat calvarial defect model and a nude mice model. In this study, β-TCP was used as the scaffold due to its highly biocompatible and resorbable properties [43, 44]. Micro-CT scanning of the rat calvaria showed that ETV2 enhanced ossification in the defect area. Meanwhile, the quantitative analyses of BMD, BV/TV, and Tb.Th parameters demonstrated that more new bone formation was detected in the ETV2-Dox (+) group, as compared with the other 3 groups. Consistently, the histological examination of samples from murine models showed that animals receiving ETV2 overexpression treatment exhibited the highest percentage of newly formed bone among the 4 groups. Collectively, our data indicated that ETV2 overexpression is positively correlated with bone formation in vivo.

## Conclusion

In summary, we found for the first time that ETV2 overexpression enhances the osteogenic differentiation of hDPSCs both in vitro and in vivo, at least partly by the activation of the ERK/MAPK and PI3K-Akt signaling pathways. These results thus indicate that ETV2 may be a promising target for treatment of cranio-maxillofacial bone defects. However, whether other signaling pathways are involved in the regulation of osteogenesis need to be further elucidated.

## Supplementary Information

**Additional file 1**: **Figure S1**. The mRNA level of CBFA1 in qRT-PCR (**a**) and RNA-Seq (**b**). *P < 0.05.

**Additional file 2**: **Figure S2.** Results of western blot analysis showed that ETV2 overexpression induced the the activation of p-ERK and p-AKT at days 3, 7 and 14 of osteogenic differentiation. *P < 0.05, compared with the 0 day group.

**Additional file 3**: **Figure S3.** HE (scale bar = 200 μm) and Masson (scale bar = 50 μm) staining in the rat calvarial defect model (n = 6).

**Additional file 4**: **Figure S4.** HE (scale bar = 50 μm) and Masson (scale bar = 50 μm) staining were performed to evaluate ectopic bone formation in nude mice (n=4).

## Data Availability

All data and materials generated or used during the study are available from the corresponding authors upon reasonable request.

## Abbreviations

MSCs: Mesenchymal stem cells
hDPSCs: Human dental pulp stem cells
BMSCs: Bone marrow mesenchymal stem cells
ETS: E26 transformation-specific
ETV2: Ets variant 2
ALP: Alkaline phosphatase
qRT-PCR: Quantitative real-time polymerase chain reaction
GAPDH: Glyceraldehyde-3-phosphate dehydrogenase
PBS: Phosphate-buffered saline
ARS: Alizarin Red S
RNA-Seq: RNA-sequencing
GO: Gene ontology
KEGG: Kyoto Encyclopedia of Genes and Genomes
BMD: Bone mineral density
BV/TV: Bone volume/total volume
Tb.Th: Trabecular thickness
HE: Hematoxylin and eosin
