# Supplementary figures and images for: ETV2 promotes osteogenic differentiation of human dental pulp stem cells through the ERK/MAPK and PI3K-Akt signaling pathways
Source: Stem Cell Res Ther. 2022 Oct 4;13:495. doi: 10.1186/s13287-022-03052-2 (PMC9533526; doi:10.1186/s13287-022-03052-2)

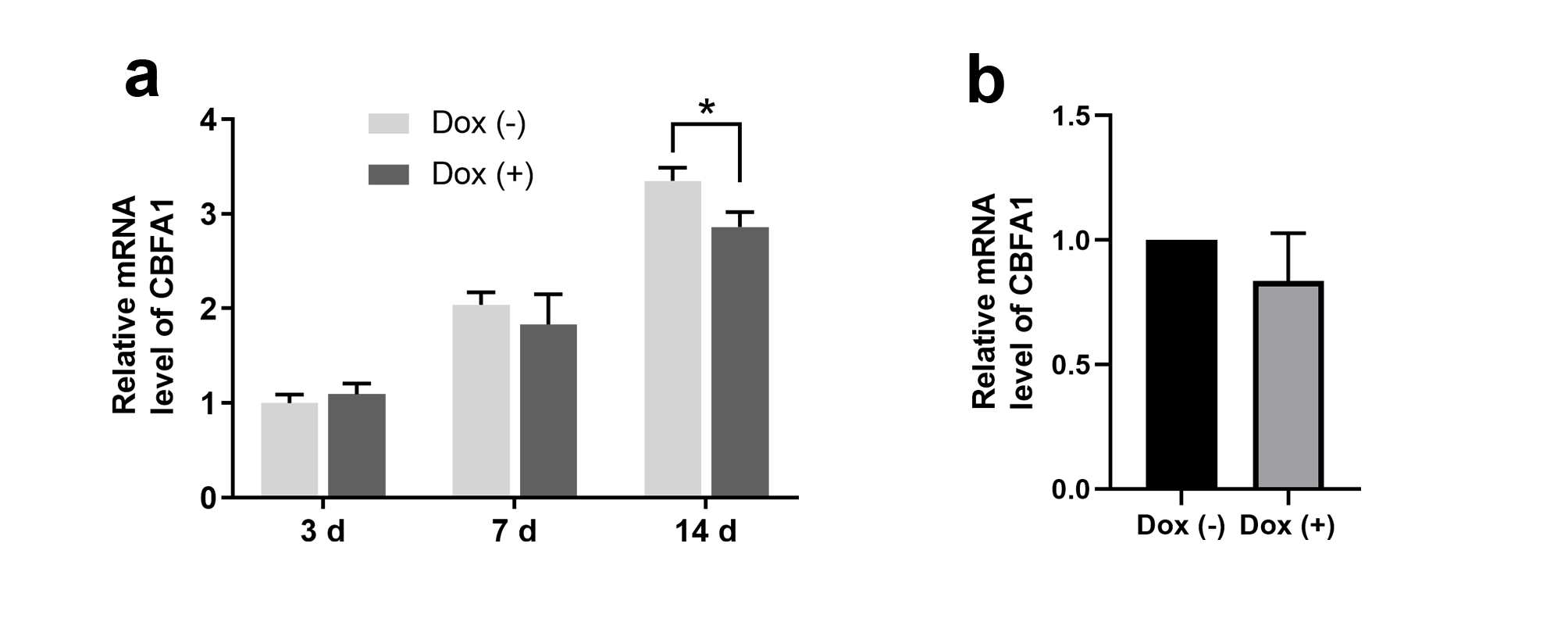

Supplement: Supplementary file 1 — Additional file 1: Figure S1. The mRNA level of CBFA1 in qRT-PCR (a) and RNA-Seq (b). *P < 0.05. [file 13287_2022_3052_MOESM1_ESM.tif]

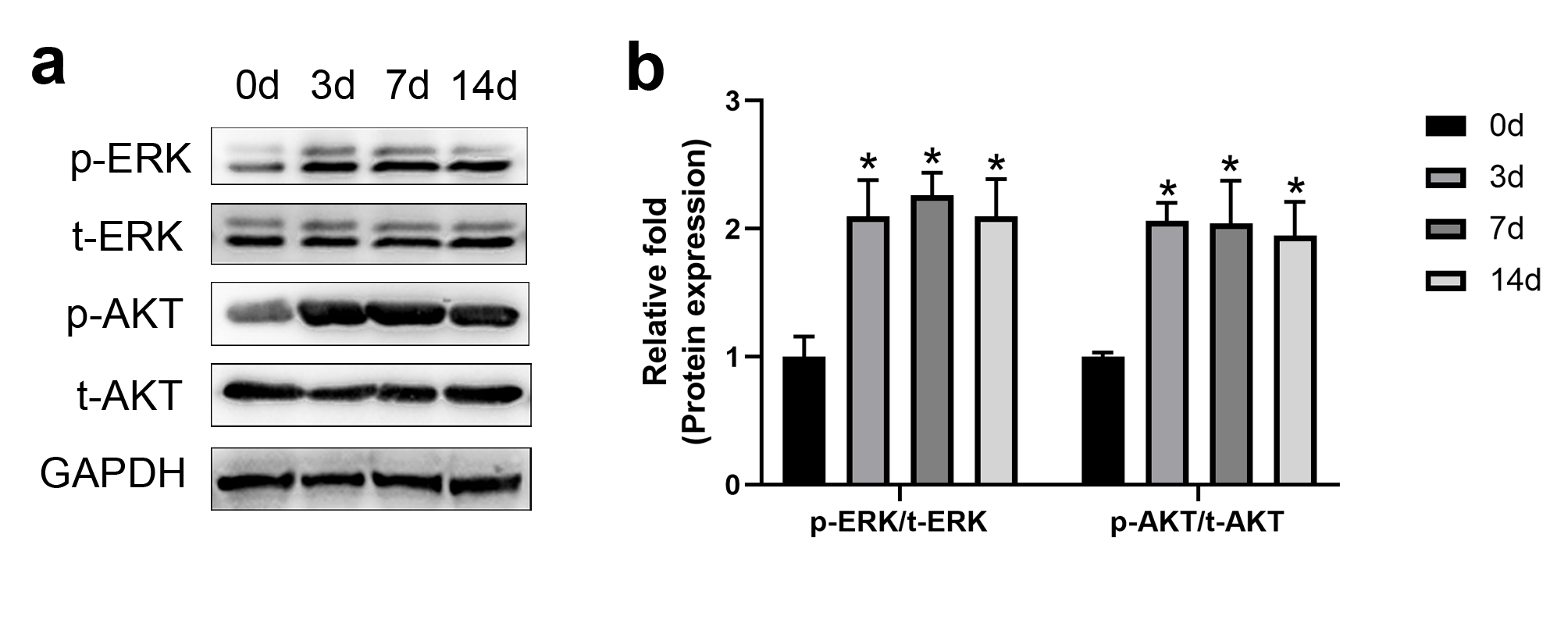

Supplement: Supplementary file 2 — Additional file 2: Figure S2. Results of western blot analysis showed that ETV2 overexpression induced the the activation of p-ERK and p-AKT at days 3, 7 and 14 of osteogenic differentiation. *P < 0.05, compared with the 0 day group. [file 13287_2022_3052_MOESM2_ESM.tif]

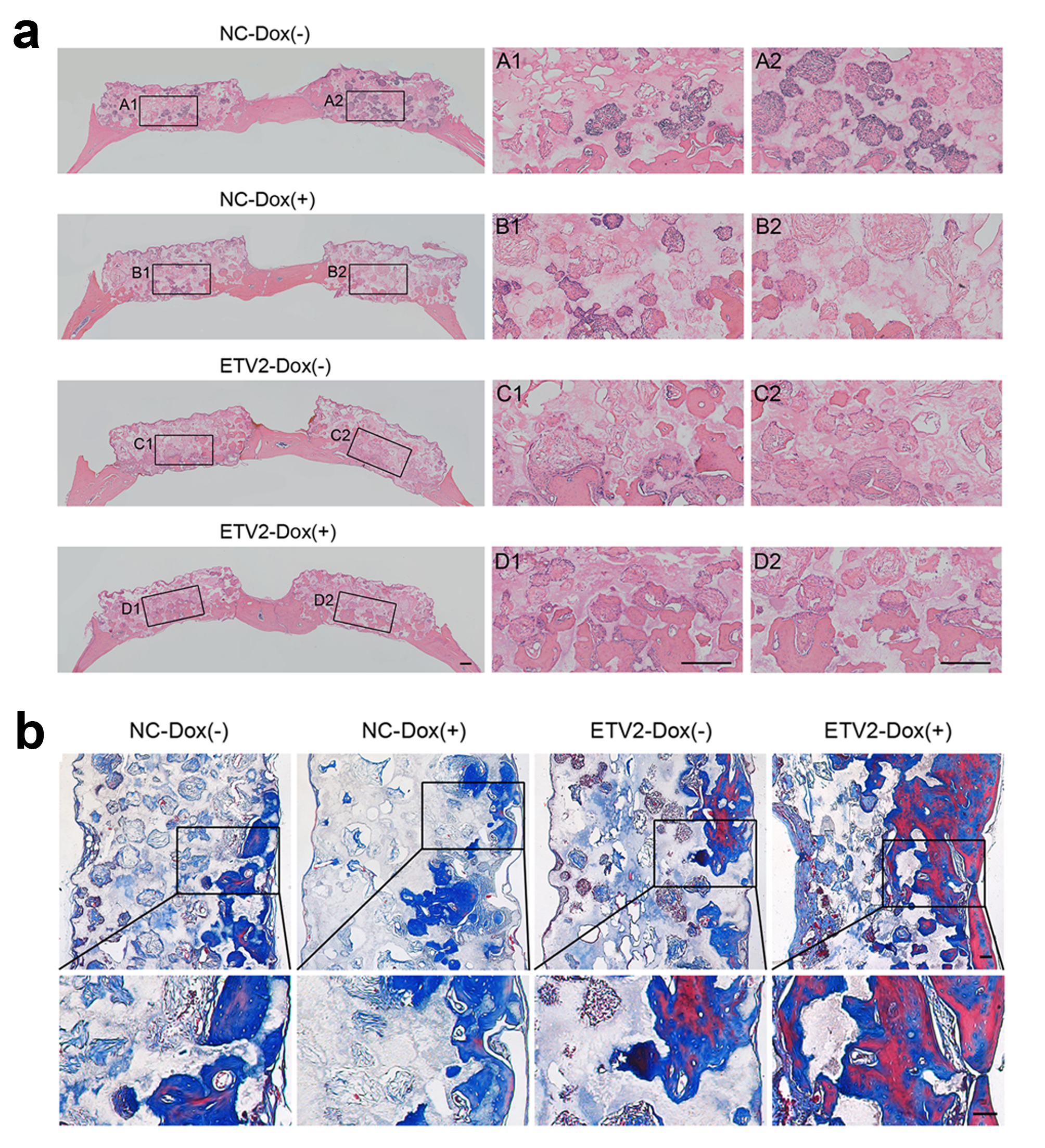

Supplement: Supplementary file 3 — Additional file 3: Figure S3. HE (scale bar = 200 μm) and Masson (scale bar = 50 μm) staining in the rat calvarial defect model (n = 6). [file 13287_2022_3052_MOESM3_ESM.tif]

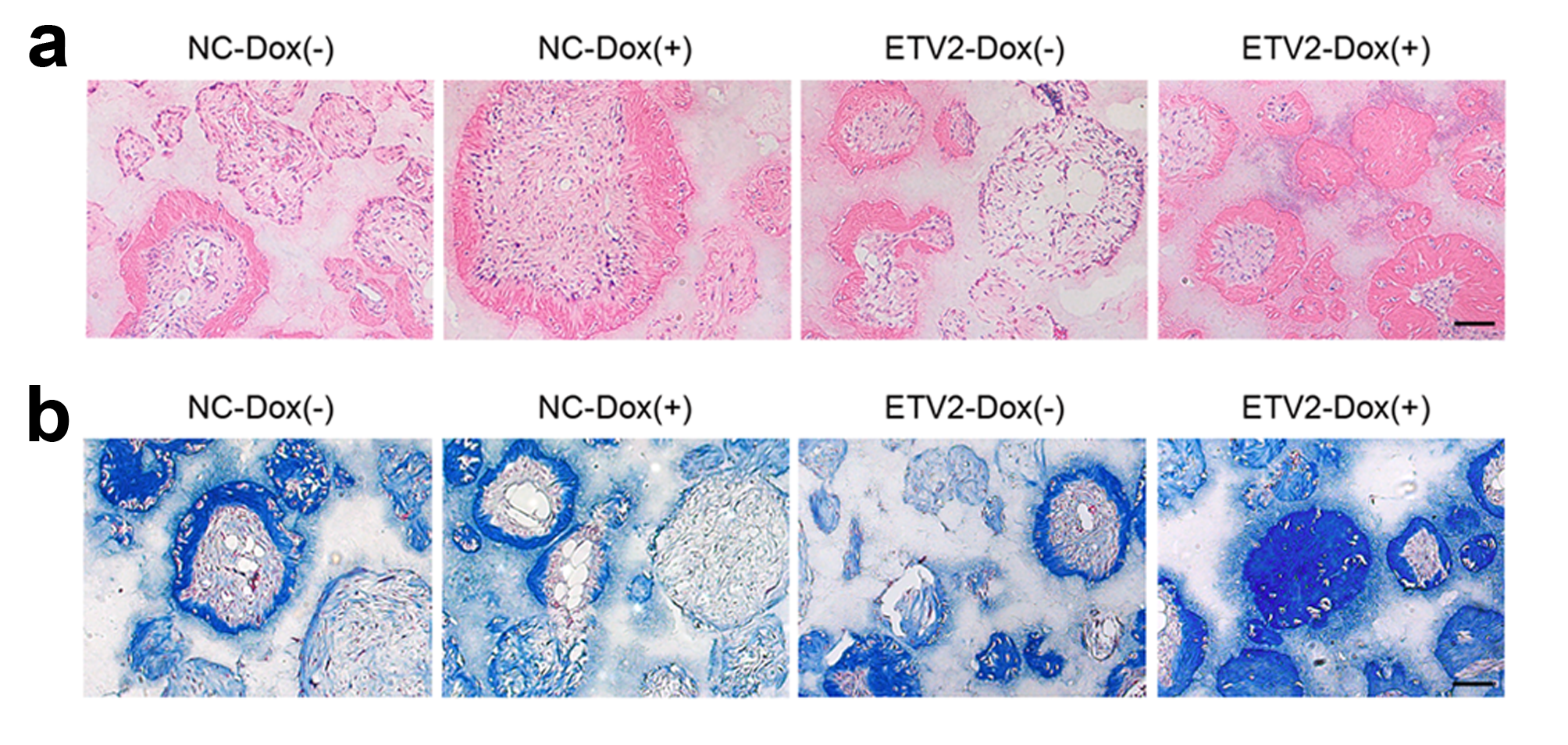

Supplement: Supplementary file 4 — Additional file 4: Figure S4. HE (scale bar = 50 μm) and Masson (scale bar = 50 μm) staining were performed to evaluate ectopic bone formation in nude mice (n=4). [file 13287_2022_3052_MOESM4_ESM.tif]
